# Attenuating effect of N-acetyl-L-cysteine against acute cocaine toxicity in rat C6 astroglial cells

**DOI:** 10.3892/ijmm.2013.1391

**Published:** 2013-05-24

**Authors:** RAMESH B. BADISA, CARL B. GOODMAN, CHERYL A. FITCH-PYE

**Affiliations:** 1 College of Pharmacy and Pharmaceutical Sciences, Florida A&M University, Tallahassee, FL 32307, USA; 2 Department of Biological Science, Florida State University, Tallahassee, FL 32310, USA

**Keywords:** acute cocaine, astroglial cells, glutathione, N-acetyl- L-cysteine

## Abstract

Astroglial cells are one of the most abundant cell types in the mammalian brain functioning in neuronal survival and in maintenance of fundamental patterns of circuitry. To date, no study has been conducted regarding the short-term impact of cocaine on these cells in cultures. The present study aimed to investigate acute cocaine (1 h) treatment on cell viability in rat C6 astroglial cells. In addition, the potential effect of N-acetyl-L-cysteine (NAC) against cocaine-induced toxicity was studied. It was observed that 1 h of acute cocaine exposure at 2, 3 and 4 mM caused a dose-dependent decrease in cell viability with an LC_50_ of 2.857 mM. Furthermore, cocaine treatment caused a decrease in glutathione (GSH) levels in the cells. It was found that cocaine did not exhibit pro-oxidant activity during its exposure to cells. Acute cocaine exposure did not induce nitric oxide (NO) release in the cells. A 5-point (1–5 mM) dose-response curve of NAC clearly indicated no adverse effect on astroglial cell viability. Pretreatment of cells with 5 mM NAC for 30 min, followed by its discard, and exposure to cocaine (2–4 mM) for 1 h protected cells against cytotoxicity by 90%. Treatment of cells with NAC-cocaine mixture rendered 100% protection. Further investigations revealed that the protection by NAC was through the increased GSH levels in the cells. Our results indicate that decreased GSH levels may represent one of the underlying pathologies of cell death and that antioxidant compounds which increase the GSH production could protect against cocaine-induced toxicity by promoting a pro-survival role in astroglial cells.

## Introduction

The abuse of drugs in modern society has increased steeply during the last decade and has become a severe psychiatric problem worldwide. Cocaine is one such widely abused and addictive drug that acts on the central nervous system (CNS). Currently, approximately 3.6 million Americans use cocaine on a regular basis (1).

The toxic effect of cocaine is observed in nearly all vital organs of the body. However, cocaine is most notably known for its psychostimulant effects on the CNS by binding to various neurotransmitter transporters with high affinity. Cocaine binding prevents the reuptake of dopamine by pre-synaptic neurons in the nucleus accumbens of the brain (2), and the excess extraneuronal dopamine at the synaptic cleft causes a euphoric feeling due to its repeated binding to post-synaptic neurons. Unfortunately, the injurious aspect of cocaine is that it causes severe toxicity to various brain cells.

To understand the mechanism of cocaine toxicity, many *in vitro* studies have been conducted. Most have been carried out for long exposure times such as 1 day (3–5) or 6 days (6) or 7 days (7). These extended endpoints may not provide an accurate picture of cocaine cytotoxicity since cocaine is removed rapidly from the body as demonstrated by its 1-h half-life (8–10). Thus, the various deleterious effects on CNS cells of cocaine users are experienced within this short period. With regards to the short half-life of cocaine, *in vitro* toxicity studies with shorter incubations will have more *in vivo* relevance in terms of understanding the cytotoxicity profile. Thus far no studies have been attempted to identify the short term impact of cocaine in different types of CNS cells.

One of the CNS cell types that is first affected by cocaine owing to their abundance is astrocytes. Since neurons depend on astrocytes for tropic support, cocaine-induced death of astrocytes may lead to neuronal dysfunction in cocaine addicts. Drugs which can prevent cocaine-induced death in astrocytes could avert neuronal dysfunction in cocaine addicts. Yet, currently there is no specific pharmacological medication available for this purpose.

In the present study, we investigated the potential role of N-acetyl-L-cysteine (NAC) against cocaine-induced toxicity in astrocytes. NAC is commonly used as a nutritional supplement for various health benefits in the US. Albeit NAC is a known antioxidant compound, its mechanism of protection in the context of a 1-h cocaine exposure has not been studied. We employed rat C6 astroglial cells in this study. These cells are astrocytes in origin and have several merits as previously outlined (11,12), making it a suitable model cell line for pharmacological studies. One-hour treatment was selected based on the 1-h half-life of cocaine (9).

## Materials and methods

### Materials

RPMI-1640, fetal bovine serum (FBS), penicillin/streptomycin sulfate, amphotericin B, phosphate-buffered saline (PBS) and L-glutamine were purchased from Mediatech (Herndon, VA, USA). Cocaine hydrochloride, crystal violet, 2′,7′-dichlorodihydrofluorescein diacetate dye (H_2_DCFDA), 2,2-diphenyl-1-picrylhydrazyl, ethylenediaminetetraacetic acid (EDTA), L-glutaraldehyde, NAC and trypan blue were supplied by Sigma Chemical Co. (St. Louis, MO, USA). All other routine chemicals were of analytical grade.

### Preparation of drug solutions

A known amount of NAC was dissolved in PBS as a 0.5 M stock. Various working stocks of NAC (40–200 mM) were prepared in the media and added to the cells. Cocaine stock and working stocks were prepared in PBS as previously described (13) just prior to the studies and added to the cells to achieve 2 to 4 mM.

### Cell culture studies

The CNS-derived rat C6 astroglial cell line (CCL-107) was purchased from the American Type Culture Collection (Rockville, MD, USA) and maintained as an adherent monolayer culture in complete RPMI-1640 (modified) medium, 2 mM L-glutamine, 10% (v/v) FBS, 100 U/ml penicillin, 100 μg/ml streptomycin sulfate and 0.25 μg/ml amphotericin B. Cells were grown in a humidified atmosphere of 95% air, 5% CO_2_ at 37°C in an incubator, and subcultured twice a week. For the cytotoxic studies, the culture was harvested by treatment with 0.05% EDTA in PBS for 2 min or less, resulting in a single-cell suspension. The cell count was assessed by 0.4% trypan blue dye exclusion assay using a hemocytometer under a light microscope.

### Treatment of cells

Treatments were performed in 96-well microtiter plates. The cells were seeded at a starting density of 2×10^4^ cells/well in a total volume of 195 μl growth medium supplemented with 10% FBS. The cells were then allowed to adhere to the wells in the incubator prior to drug exposure. Cells which were typically ~80–90% confluent were treated with 2, 3 and 4 mM cocaine in a final volume of 5 μl. In some experiments, 5 μl NAC from different working stocks was added per well to achieve the final concentrations of 1 to 5 mM. Controls and the treated samples were always present in different wells of the same 96-well microtiter culture plate. These plates were incubated for 1 h in an incubator in 5% CO_2_ at 37°C.

### Evaluation of cytotoxicity

At the end of the 1-h treatment, 100 μl of 0.25% glutaraldehyde was added per well and incubation was carried out for 30 min to fix the cells to the polystyrene surface of the culture plates. The plates were gently washed three times and air dried. The cells were then stained with crystal violet as described previously (14,15). The dye in each well was extracted with 100 μl of 50 mM sodium phosphate monobasic solution, containing 50% ethyl alcohol. The plates were gently vortexed, and the optical density (OD) measurements of the incorporated dye in the viable cells were carried out at 540 nm using a microplate reader (BioTek Instruments, Inc., Wincoski, VT, USA). From the treated and control absorbance values, the percentage of cells killed was determined using the following equation: [1 − (T/C)] × 100, where T is the average absorbance values of the treated cells, and C is the average absorbance values of the control cells.

### Antioxidation activity of NAC

The experiment was carried out at different final concentrations of NAC (0.02–0.1 mM) in 1-ml Eppendorf tubes without cells with ethanol as a solvent. Free radical 2,2-diphenyl-1-picrylhydrazyl was added to the samples at a final concentration of 0.1 mM. Milk thistle, a known antioxidant, was used as a positive control (0.1 mg/ml). After a 30-min incubation at room temperature, the solution in each tube was transferred to a 96-well plate. Absorbance at 517 nm was read using a microplate reader. The percentage of scavenging activity was calculated (16) by the following equation: [(OD of control - OD of NAC)/OD of control] × 100.

### Cocaine pro-oxidant study

The pro-oxidant activity of cocaine was assessed in phenol red-free RPMI-1640 (modified) media in 96-well microtiter plates without employing the cells. The assay utilized H_2_DCFDA dye. Different concentrations of cocaine (final 2–4 mM) were incubated with 0.1 mM (final) H_2_DCFDA for 1 h in an incubator with the plates capped in a normal fashion. The plates were read with the excitation filter set at 485 nm and the emission filter at 530 nm in a Microplate Fluorometer Model 7620, version 5.02 (Cambridge Technology, Inc., Watertown, MA, USA). Hydrogen peroxide (0.1 mM) was used as a positive control.

### Nitric oxide (NO) assay

NO production with cocaine treatment (2–4 mM) was assessed as reported previously (17) in the media lacking phenol red. A 5-point standard curve was generated by using various concentrations of sodium nitrite (25–400 μM) in culture media. The absorbance readings at 546 nm were measured using a microplate reader.

### Estimation of cellular glutathione (GSH) levels

Cellular GSH was determined according to Smith *et al*(18). In brief, at the end of the incubation, the cells were deproteinized with 2% 5-sulfosalicylic acid (10 μl/well) for 30 min at 37°C, followed by addition of 90 μl of the reaction mixture containing 0.416 mM sodium EDTA, 0.416 mM nicotinamide adenosine dinucleotide phosphate (NADPH), 0.835 mM 5,5-dithiobis-2-nitrobenzoic acid (DTNB), 0.083 mM sodium phosphate buffer, pH 7.5. The mixture was incubated at 37°C for 30 min, and the absorbance was measured at 412 nm using a plate reader.

### Pretreatments with NAC

Experiments were performed in 96-well culture plates under the conditions described above in complete media. In brief, the C6 astrocytes were pretreated with 5 μl of 0.2 M NAC to obtain a final concentration of 5 mM for 30 min. The media was discarded, and the cells were washed gently to remove NAC. Then 195 μl fresh media were added per well, followed by treatments with 5 μl of 80–160 mM cocaine working stocks to achieve final concentrations of 2–4 mM for 1 h. In the case of NAC-cocaine mixture studies, 0.2 M NAC was incubated with 80–160 mM cocaine in Eppendorf tubes for 30 min in an incubator at 37°C and added to the cells to achieve final concentrations of 5 mM NAC and 2, 3 and 4 mM cocaine. At the end of the incubation, cell viability and GSH levels were determined as described above.

### Statistical analysis

The experimental results are presented as means ± standard error of the mean (SEM). The data were analyzed for significance by one-way ANOVA and then compared by Dunnett’s multiple comparison tests using GraphPad Prism Software, version 3.00 (GraphPad Software, Inc., San Diego, CA, USA). The test value of P<0.05 was considered significant. The LC_50_ or ED_50_ values representing the millimolar concentration of cocaine needed to show a 50% response were determined from the graphs (19).

## Results

### Dose-response study with NAC

**Figure 1:**
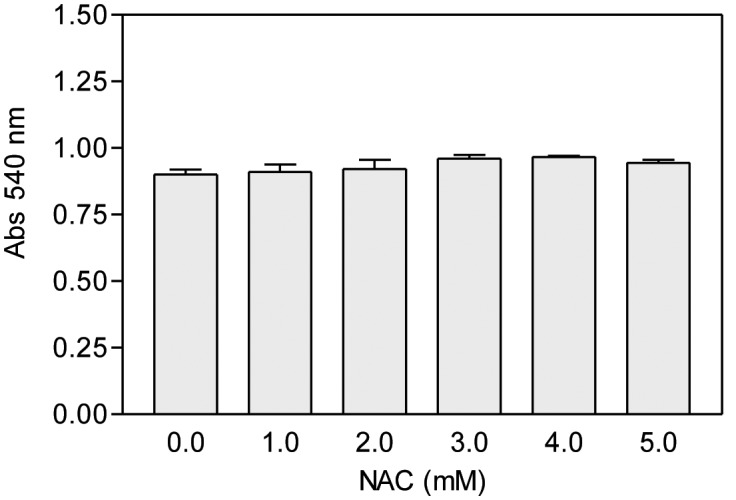
Effect of NAC on rat C6 astroglial cell viability. The cells with an initial density of 2×10^4^ were treated with various concentrations of NAC for 1 h. Data are represented as means ± SEM (n=6, ^*^P>0.05, insignificant from control).

**Figure 2:**
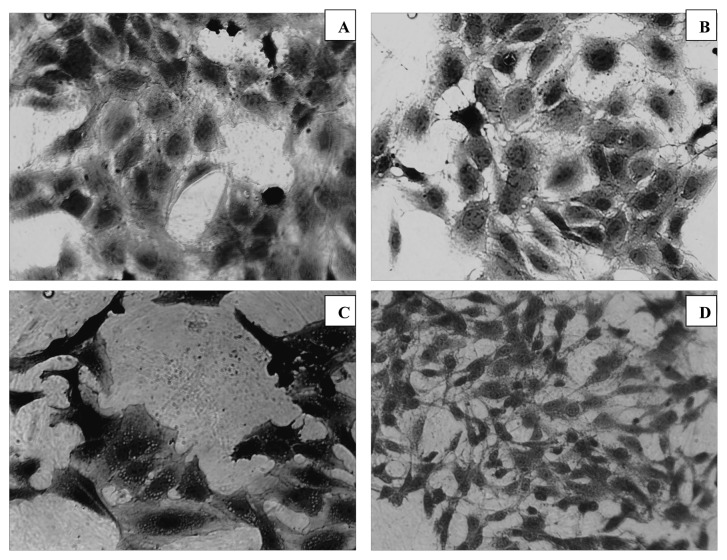
Morphological features of the astroglial cells. (A) Cells were treated with PBS or (B) 5 mM NAC for 1 h or (C) 2 mM cocaine for 1 h or (D) pretreated with 5 mM NAC for 30 min, followed by its discard and treatment with 4 mM cocaine for 1 h. Crystal violet-stained cells were photographed under an inverted phase contrast 1X-70 Olympus microscope with ×40 objective.

Prior to evaluating any pro-survival action of NAC on C6 astroglial cells against acute cocaine treatment, we first assessed NAC effects on cell viability at concentrations of 1, 2, 3, 4 and 5 mM. The treatment was continued for 1 h, and the data are shown in Fig. 1. It was observed that NAC did not cause cell death (P>0.05, n=6) at any concentration compared to the control (Fig. 1). The viability results were corroborated by the microscopic observations of crystal violet-stained cells. The photomicrographs revealed that the morphology of the NAC-treated cells was similar in resemblance to the untreated control cells (Fig. 2B). Lack of NAC toxicity in astroglial cells corroborated a similar viability result with ascorbic acid as previously reported (20).

### Scavenging activity

**Figure 3:**
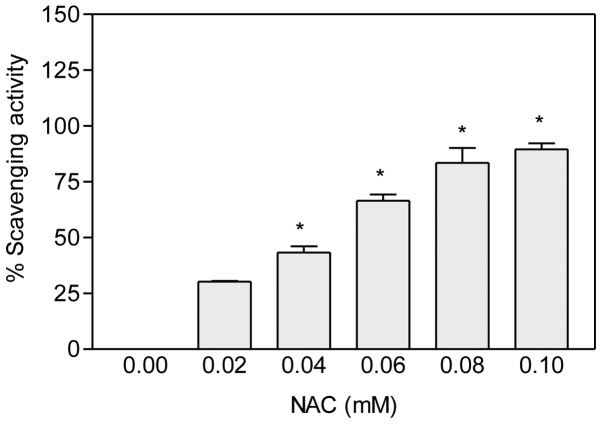
Scavenging activity. Various concentrations of NAC were added to 1-ml Eppendorf tubes without cells with ethanol as a solvent, and incubated in 0.1 mM 2,2-diphenyl-1-picrylhydrazyl-free radical for 30 min. Data are represented as means ± SEM (n=9, ^*^P<0.01, highly significantly different from the control).

NAC is a well-known antioxidant. In this study, we aimed to determine its activity under our experimental conditions. Initial studies with concentrations ranging between 0.1 and 1 mM NAC showed high antioxidation activity. In the subsequent experiments, the concentrations were adjusted between 0.01 and 0.1 mM. The data are presented in Fig. 3. A significant (P<0.01, n=9) dose-dependent increase in the scavenging activity of NAC was noted. The ED_50_ value was found to be 0.047 mM.

### Pro-oxidant activity of cocaine

**Figure 4:**
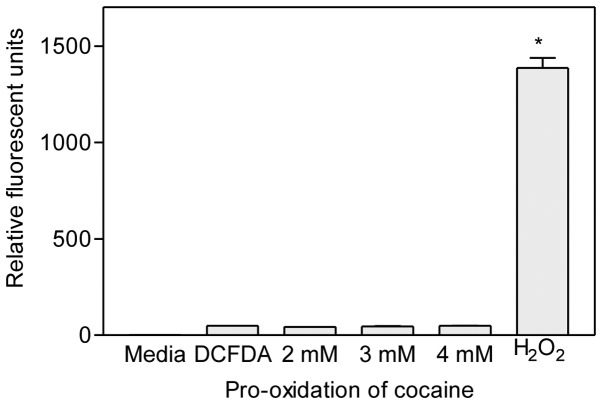
Determination of pro-oxidation activity of cocaine. Various concentrations of cocaine (2–4 mM) were incubated in 0.1 mM H_2_DCFDA dye for 1 h at 37˚C. Data are represented as means ± SEM (n=6, ^*^P>0.05, insignificant from the control)

Various concentrations of cocaine (final 2–4 mM) were mixed with H_2_DCFDA dye (final 0.1 mM) and incubated without cells for 1 h at 37°C. Since this dye is converted to fluorescent dichlorodihydrofluorescein (DCF) molecules upon oxidation by reactive oxygen species (ROS), it represents a convenient method to determine whether cocaine exhibits pro-oxidant activity during treatment periods in cells. Results indicated that there was no progressive increase (P>0.05, n=6) in the amount of fluorescence from the dye following cocaine treatment at any concentration (Fig. 4). These results clearly indicate that cocaine did not exhibit pro-oxidant activity. In contrast, 0.1 mM H_2_O_2_, the positive control, released very high levels of fluorescence from the H_2_DCFDA dye, demonstrating the reliability of the method.

### Lack of NO release

**Figure 5:**
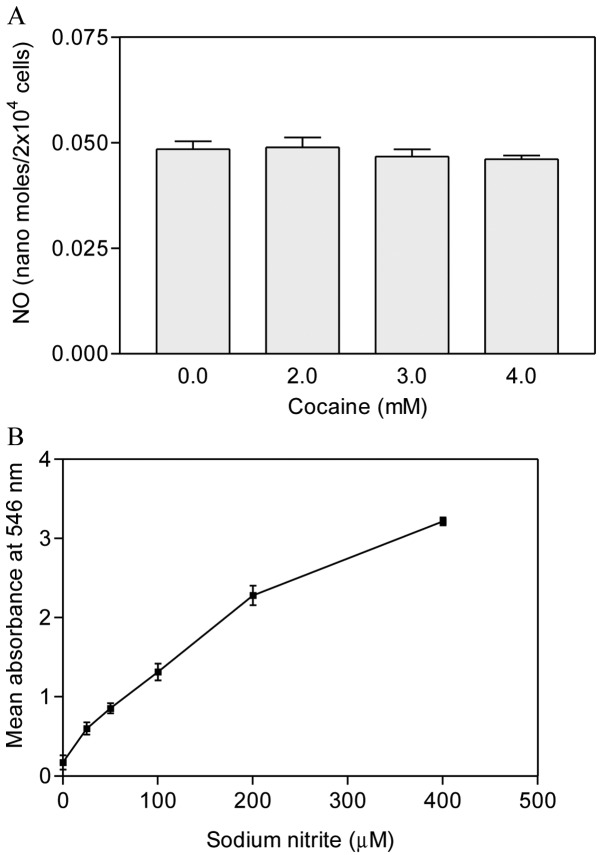
(A) Nitric oxide production in cocaine-treated astroglial cells. The cells were seeded in 96-well plates with complete RPMI-1640 media lacking phenol red, containing 10% FBS and treated with 2, 3 and 4 mM cocaine for 1 h. Nitric oxide (NO) was detected with Griess reagent. Data are presented as means ± SEM (n=12, ^*^P>0.05, insignificant in comparison to the control). (B) Standard curve of sodium nitrite (25–400 μM).

Overproduction of NO contributes to various pathological conditions in the CNS. In order to study the role of cocaine in NO production, the C6 astrocytes were treated with cocaine (2–4 mM) for 1 h. The cells were not challenged with lipopolysaccharide or interferon-γ as these cells contain NO synthase (21) and our objective was to determine whether cocaine induces this enzyme. It was observed that acute cocaine did not induce (P>0.05, n=12) cellular NO synthase for NO production when compared to the controls (Fig. 5A). On the other hand, sodium nitrite standard exhibited a linear response (Fig. 5B).

### Attenuation of cocaine toxicity by NAC

**Figure 6:**
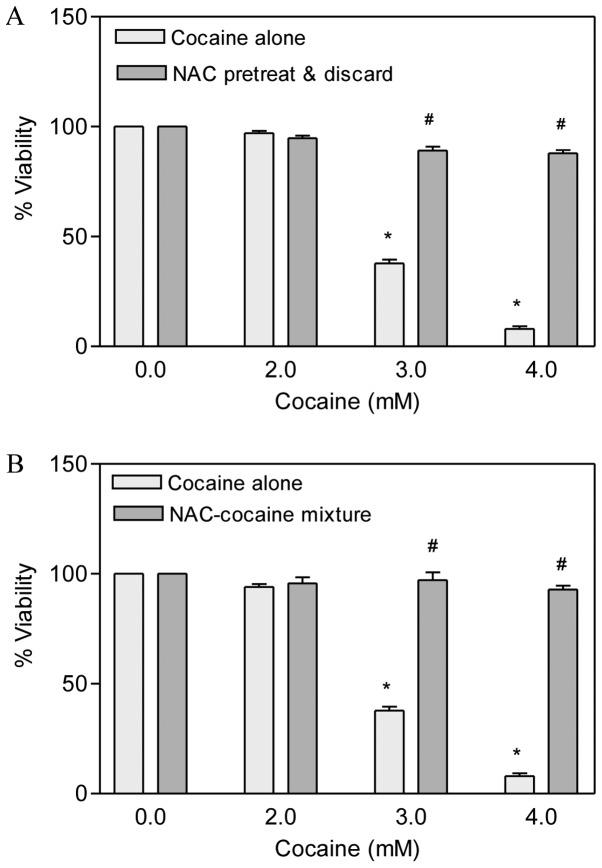
Attenuating action of NAC against cocaine-induced toxicity. Cells were pretreated with 5 mM NAC for 30 min, followed by its discard, and then treated with (A) cocaine for 1 h or (B) NAC-cocaine mixture for 1 h. Data are represented as means ± SEM (n=16), ^*^P<0.01, significant in comparison the control; ^#^P<0.01, significant difference between non-pretreated and pretreated cocaine-exposed cells.

Based on the non-toxicity of NAC to the C6 astrocytes (Fig. 1), we selected 5 mM NAC to determine its efficacy for cell protection against cocaine-induced toxicity. For this purpose, the cells were initially pretreated with NAC at a final concentration of 5 mM for 30 min. Then the media were discarded completely, and cells were re-fed with fresh media, followed by treatment with acute cocaine at 2, 3 and 4 mM for 1 h. The cell viability results indicated that NAC rendered 90–100% protection (P<0.01, n=16) against cocaine cytotoxicity (Fig. 6A). The LC_50_ value was found to be >4 mM cocaine. In the absence of NAC pretreatment, a significant (P<0.01, n=16) dose-dependant cell death by cocaine was noted with an LC_50_ value of 2.857 mM. The cell morphology of pretreated cells following cocaine exposure remained the same as that of the control cells (Fig. 2D). In contrast, the cell morphology of the non-pretreated cells with cocaine was altered significantly (Fig. 2C). In addition, several cytoplasmic vacuoles were observed in these cells. In the case of the NAC-cocaine mixture studies, the cell viability remained 100% (P<0.01, n=16) in the presence of NAC, while in its absence, the viability was significantly decreased (P<0.01, n=16) (Fig. 6B). Thus, either pretreatment with NAC followed by cocaine or co-treatment of NAC and cocaine produced very similar results (Fig. 6).

### NAC pretreatment enhances GSH synthesis

**Figure 7:**
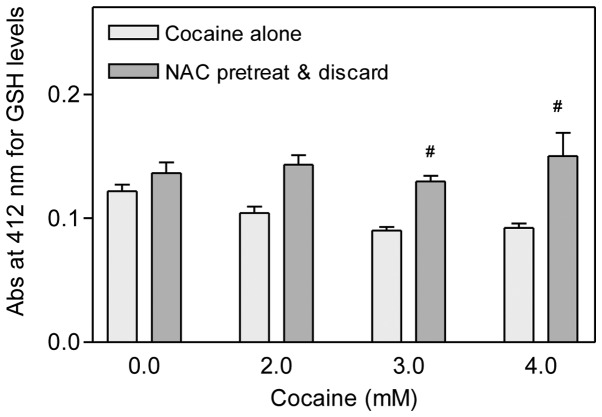
Increased glutathione (GSH) levels of NAC pretreatment. The cells were pretreated with 5 mM NAC for 30 min, followed by its discard, and then treated with cocaine for 1 h. Data are represented as means ± SEM (n=8), ^#^P<0.01, significant difference between non-pretreated and pretreated cocaine-exposed cells.

Based on the significant results of cell protection by NAC pretreatment against cocaine exposure (Fig. 6), we subsequently evaluated the GSH levels in these cells. In the non-pretreated cells, cocaine treatment at 2, 3 and 4 mM decreased the GSH levels (Fig. 7). However, with a 30-min pretreatment with 5 mM NAC, followed by its discard, the GSH levels were increased significantly (P<0.05, n=8) in all treated cells.

## Discussion

Cocaine is hydrolyzed rapidly by various esterases in the body (22). In addition, it is also degraded non-enzymatically by auto-oxidation at basic physiological pH (23). In spite of these losses, entry of a small fraction of intact cocaine into various domains of the brain can cause cell death or deformation of cell morphology to a high extent. In the present study, cocaine treatment of astroglial cells was evidenced by the presence of cytoplasmic vacuoles (Fig. 2C). Previous studies showed that cocaine interaction decreased both membrane potential (13) and the general respiratory status (17) of mitochondria. This type of association often results in high ROS release in cells (24). It is possible that a portion of the released ROS accounts for cocaine’s pro-oxidation action. We employed a fluorometric method to detect this activity. The data clearly indicated that cocaine did not have pro-oxidation activity (Fig. 4) during the study period. Thus, the generated ROS was solely due to cocaine interaction with mitochondria.

Cocaine interaction with mitochondria also creates a condition similar to hypoxia. This situation in astroglial cells favors inflammation and causes excess release of NO by inducible NO synthase (25). Dysfunctional mitochondria (26) and excess release of NO (27) have been implicated in several late-onset neurodegenerative diseases such as Parkinson’s disease (28), schizophrenia and Alzheimer’s disease (29). There are some speculations that drug addicts are more likely to be affected by these diseases at an early age. Unfortunately, few reports are available to link the release of NO from astroglial cells in drug addicts to neurodegenerative diseases. Our data clearly rule out that astroglial cells are the source of NO release (Fig. 5). However, it may still be possible that NO is released from different CNS cells such as neurons or activated microglial cells following cocaine exposure. Lack of NO production in our study suggests that cocaine toxicity was not through the NO pathway in astroglial cells.

It is known that cocaine induces damage to different types of CNS cells. Of the three main types of cells found in the brain, namely neurons, astroglial cells and oligodendrocytes, astrocytes are the most abundant cells (30). These cells not only function in neuronal survival but also maintain the fundamental patterns of circuitry. Furthermore, due to close contacts with endothelial cells of the blood-brain barrier (BBB) (31), it is possible that astrocytes are the first cells to face toxic insults by cocaine. Since neuronal survival depends on astrocytes, death of astrocytes by cocaine could immediately trigger neuronal dysfunction. This may lead to cognitive defects, an observation commonly found in drug addicts. Counter measures, which inhibit cocaine-induced death in astrocytes, may prevent eventual neuronal dysfunction. In the absence of timely remedial measures, it is possible that a strong stage may be set for long-term effects such as neuronal death or drug tolerance in cocaine addicts. Therefore, the main question is how to decrease or prevent the toxic effects of cocaine in cells.

To this end, we explored the effects of the pretreatment of cells with antioxidants in cultures. We evaluated the role of NAC, a sulfhydryl compound, against acute cocaine-induced toxicity in C6 astroglial cells. The antioxidant activity of NAC is well known (32). Prior to testing its attenuating effect, a dose-response study was conducted to determine the optimum dose for further studies. The non-toxicity of NAC following a 1-h exposure to astroglial cells was evident in the viability study (Fig. 1). Even at a dose of 5 mM, all cells were alive and maintained the same morphological features as the untreated controls (Fig. 2A and B). Thus, we pretreated the astroglial cells with 5 mM NAC.

We observed that a 30-min NAC pretreatment decreased the cocaine toxicity by 90% in the cells (Fig. 6A). Furthermore, the cells treated with NAC-cocaine mixture were rendered 100% protection (Fig. 6B). Our study demonstrated that cocaine exposure caused a decrease in the GSH levels in cells. However, NAC pretreatment boosted these levels similar to the controls even in the presence of cocaine (Fig. 7). It is known that hydrolysis of NAC provides an L-cystein precursor for GSH biosynthesis in cells. Comparison of the controls indicated that there was a clear insignificant increase in GSH level in the NAC-pretreated cells than that in the non-pretreated cells (Fig. 7). This observation suggests that the L-cystein precursor from hydrolyzed NAC was utilized in intracellular GSH synthesis during the pretreatment period. These results clearly suggest that the observed cell protection against cocaine-induced toxicity was through increased GSH levels. The results of cocaine toxicity and NAC protection appear to imply that both compounds share similar target sites in these cells. Another important finding in our study was that we observed no cell death following NAC-cocaine co-treatment (Fig. 6B). We plan to use our treatment *in vivo* to determine whether these same protective effects are observed in animals.

## Abbreviations

BBB: blood-brain barrier
CNS: central nervous system
DCF: dichlorodihydrofluorescein
DTNB: 5,5-dithiobis-2-nitrobenzoic acid
EDTA: ethylene diamine tetraacetic acid
FBS: fetal bovine serum
GSH: glutathione
H_2_DCFDA: 2′,7′-dichlorodihydrofluorescein diacetate dye
NAC: N-acetyl-L-cysteine
NADPH: nicotinamide adenosine dinucleotide phosphate
NO: nitric oxide
OD: optical density
PBS: phosphate-buffered saline
ROS: reactive oxygen species
